# Prognosis of Old Intensive Care COVID-19 Patients at a Glance: The Senior COVID Study

**DOI:** 10.5152/TJAR.2022.21321

**Published:** 2022-04-01

**Authors:** Charles-Hervé Vacheron, Laurent Bitker, Fabrice Thiolliére, Fabien Subtil, Paul Abraham, Vincent Collange, Baptiste Balança, Max Haïne, Céline Guichon, Christophe Leroy, Marie Simon, Amélie Malapert, Mélanie Roche, Jean-Baptiste Pialat, Laurent Jallades, Alain Lepape, Arnaud Friggeri, Claire Falandry

**Affiliations:** 1 Department of Anaesthesiology and Reanimation, Centre Hospitalier Lyon Sud, Hospices Civils de Lyon, Lyon, France; 2 Biometrics and Evolutionary Biology Laboratory, Biostatistics-Health Team, Hospices Civils de Lyon, Villeurbanne, France; 3 Division of Public Health, Department of Biostatistics and Bioinformatics, Lyon, France; 4 Department of Intensive Medicine and Resuscitation, Croix Rousse Hospital, Hospices Civils de Lyon, Lyon, France; 5 Department of Anaesthesia and Resuscitation, Édouard Herriot Hospital, Hospices Civils de Lyon, Lyon, France; 6 Claude Bernard University Faculty of Medicine, Lyon, France; 7 Medipole Lyon Villeurbanne Hospital, Villeurbanne, France; 8 Department of Anaesthesia and Resuscitation, Pierre Wertheimer Hospital, Hospices Civils de Lyon, Lyon, France; 9 North West Hospital of Villefranche, France; 10 Inter-University Laboratory of Human Movement Biology, Villeurbanne, France; 11 Department of Anaesthesia and Surgical Resuscitation, Croix Rousse Hospital, Hospices Civils de Lyon, Lyon, France; 12 Department of Intensive Care Medicine, Emile Roux Hospital Center, le Puy en Velay, France; 13 Plateforme IC-HCL, Centre Hospitalier Lyon Sud, Hospices Civils de Lyon, Lyon, France; 14 Department of Biological Hematology, Lyon Sud Hospital South Biology and Pathology Center, Pierre-Bénite, France; 15 Department of Intensive Care, Henry Gabrielle Hospital, Hospices Civils de Lyon, Lyon, France; 16 Department of Critical Care, Lyon Sud Hospital, Hospices Civils de Lyon, Lyon, France; 17 Division of Geriatrics, Center Hospitalier Lyon Sud, Hospices Civils de Lyon, Lyon, France

**Keywords:** Coronavirus disease 2019, ethical, mortality, nomogram, prognostic factors, statistical modeling

## Abstract

**Objective:**

Admission in the intensive care unit of the old patient with coronavirus disease 19 raises an ethical question concerning the scarce resources and their short-term mortality.

**Methods:**

Patients aged over 60 from 7 different intensive care units admitted between March 1, 2020 and May 6, 2020, with a diagnosis of coronavirus disease 19 were included in the cohort. Twenty variables were collected during the admission, such as age, severity (Simplified Acute Physiology Score [SAPS] II), several data on physiological status before intensive care unit comorbidities, evaluation of autonomy, frailty, and biological variables. The objective was to model the 30-day mortality with relevant variables, compute their odds ratio associated with their 95% CI, and produce a nomogram to easily estimate and communicate the 30-day mortality. The performance of the model was estimated with the area under the receiving operating curve.

**Results:**

We included 231 patients, among them 60 (26.0%) patients have died on the 30th day. The relevant variables selected to explain the 30-day mortality were Instrumental Activities of Daily Living (IADL) score (0.82 [0.71-0.94]), age 1.12 (1.07-1.18), SAPS II 1.05 (1.02-1.08), and dementia 6.22 (1.00-38.58). A nomogram was computed to visually represent the final model. Area under the receiving operating curve was at 0.833 (0.776-0.889).

**Conclusions:**

Age, autonomy, dementia, and severity at admission were important predictive variables for the 30-day mortality status, and the nomogram could help the physician in the decision-making process and the communication with the family.

**Main Points**

- Admission in the intensive care unit of the coronavirus disease 19 among the elderly raises several ethical questions.
- A scoring system including the IADL, SAPS II, presence of dementia, and age is available with good discrimination to predict the 30-day mortality.
- A nomogram could help the physician in the decision-making process and the communication with the family.

## Introduction

During the coronavirus disease period, the ethical question of the criteria for admission to the intensive care unit was a major issue.^1^ The problem with the critical care and the old patients is already the subject of numerous studies which suggest that the criteria should not be based on age but on frailty and dependence.^2^ The old patients are more often victims of COVID and at risk of developing severe forms. In a context of limited or strained resources, the decision to admit a patient to the intensive care unit (ICU) can be a particularly difficult moment for the clinician.^3^ In particular, for patients requiring mechanical ventilation, in whom the reported mortality exceeds 40%, can we allocate a scarce resource to patients with a poor prognosis^4^? 

Indeed, the COVID-19 stay into ICU is not only associated with a high mortality^4^ but with a long length of stay and prolonged rehabilitation. The chance to recover a good quality of life becomes therefore unlikely for frail patients.^5^ The decision to admit a patient in the ICU results in a complicated balance between previous clinical status, the severity of the illness, and probability of short-term survival, leading to complex ethical decision.^6^ Moreover, communication tools for the patient or close relative available to the clinician are currently lacking in this time of the pandemic, despite their acknowledge benefit.^7^

Our objective was to model the mortality among old patients admitted to ICU with a COVID-19 diagnosis, according to available data at admission (autonomy, comorbidity, severity of the illness, biological value) and to propose simple and useful decision-making and communication tool to predict 30-day mortality into the ICU. 

## Methods

A multicenter observational cohort study was conducted on the patients admitted in the ICU between March 1, 2020, and May 6, 2020. The study was funded by the Hospices Civils de Lyon. Seven French ICUs were involved in the recruitment of patients. 

The study protocol (V1.0 of April 7, 2020) was approved by a COVID-19-dedicated Ethics Committee of the Hospices Civils de Lyon on May 12, 2020, and declared on the ClinicalTrials platform on June 9, 2020 (NCT04422340), with the protocol published elsewhere.^8^ Ethical approval according to French law was such that formal written consent from participants was not required, but individual information explaining the study was given to the patient. Depending on the patients’ clinical condition, a waiver request was justified in accordance with the International Council for Harmonisation of Technical Requirements for Pharmaceuticals for Human Use Guideline for Clinical Practice. 

Inclusion criteria were patients admitted with a laboratory or radiologic confirmed diagnosis of severe acute respiratory syndrome coronavirus 2 and an admission age over 60. 

After the consent of the patient or his/her caregiver to participate in the study was obtained, a questionnaire was delivered by phone to explore the functional status of the patient based on the patients’/caregiver perspective. We studied age, sex, body mass index, the previous clinical status (autonomy [IADL score], frailty [Fried’s score of at least 3 points], 1 month before COVID-19, and fall 6 months before admission in ICU), recent weight loss (loss of weight the month before hospitalization), the Cumulative Illness Rating Scale (CIRS) for the estimation of the comorbidity, and the existence of mild to moderate dementia. Other data collected included delay between symptom and ICU admission, the hospitalization prior to ICU admission, SAPS II (computed on the worst parameters in the first 24 hours), PaO_2_/FiO_2_ ratio, and biological variable at ICU admission.

The objective was to predict day 30 mortality with the variables available to the clinician during the admission for patients aged over 60.

### Statistical Analysis

Continuous variables are described by their median and interquartile range, and categorical variables are described with the number of patients associated with their percentage (n, %). Differences between groups were tested with the Wilcoxon rank-sum test, chi-square test, or Fisher’s test. 

Variables which presented more than 30% of missing variables were not included in the analysis. For the remaining variables, imputation of the missing variables was performed using the Multiple Imputation by Chained Equations (MICE) algorithm. 

Then, a bidirectional backward stepwise regression was performed to select the best model on the Bayesian information criterion (BIC). The accuracy of the final model was assessed by the area under the receiver operating curve (AUROC). 

Multivariate odds ratios (ORs) associated with their 95% CI and respective *P* value were estimated. Finally, a visual representation of the model (nomogram) was plotted to graphically illustrate the net effect of each selected covariate.

*P* values less than .05 were considered significant. Analyses were performed using R software version 3.6.4.

## Results

We included 231 patients. On the 30th day, 60 (26.0%) patients died. The 30-day non-survivors were statistically older (78 [71-82] vs 72 [66-76] years old), more likely to have dementia or to be frail. The IADL score was statistically lower among the non-survivors (8 [8-8] vs 6 [4-8]). Moreover, they had a higher number of comorbidities (CIRS 5 [2-7] vs 7 [4-11]) and a lower delay between symptom and ICU admission. They presented a higher SAPS II but no statistical difference in PaO_2_/FiO_2_ ratio. Except in creatinine value, the biological characteristics were similar between D30 survivor and D30 non-survivor. Detailed results are available in Table 1. 

### Modeling Strategy of the 30-Day Mortality

**Figure 1.**
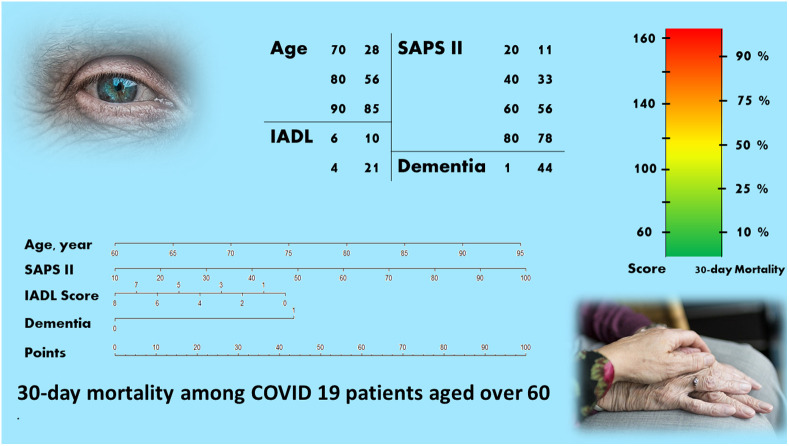
Nomogram of the logistic regression model.

We included 19 variables in the modeling strategy. Stepwise regression initially selected 3 variables: IADL score, age, and SAPS II. During stepwise regression, the variables are subsequently removed from the model from the less to the more relevant variable. The last variable eliminated from the regression was dementia, and because of its clinical relevance and low difference in BIC between the 2 models (224.6 vs 225.3), we chose to keep dementia in the final model. The AUROC of the final model was 0.833 [0.776-0.889]. The ORs of the selected variables are available in Table 2, and the nomogram derived from this model is available in Figure 1.

## Discussion

Four variables were selected in the final model for the prediction of the 30-day mortality: severity (SAPS II), age, autonomy (IADL score), and dementia. All these variables had a clinical relevance and are understandable by non-medical staff. 

The 30-day mortality observed is consistent with the literature, ranging from 20% to 40%^4,9^ (p30), and the selected prognostic factors are known to be associated with mortality. Most studies of COVID 19 do not consider the patient’s prior condition, such as frailty or independence.

Here, the interest of the nomogram is not only to compute a crude predicted mortality but also to interpret the importance of the variable, easier than the OR. Indeed, the effect of the variables on mortality is presented in the format of axe to estimate a score, and there is a visual relationship between the importance of the variable and the attributable score. 

For example, the predicted mortality of an 80 years old patient with a SAPS II score of 40, good autonomy, and no known dementia has a score of 56 + 33 + 0 + 0 = 89 points and therefore, predicted mortality of around 30%. Despite his younger age, a 70 years old patient with a slight loss of autonomy (IADL 6) and a SAPS II score of 60 and known dementia has a higher score (110) with a predicted mortality of 45%. 

Visually, we are able to observe the importance of the age and the SAPS II score for predicting the 30-day mortality and to correlate dementia to the same effect of an increase in age of 15 years or an IADL at 5 to an increase in age of 5 years old. This tool will also most certainly help the communication between the physician and the patient or their family to explain the decision that has been taken. It requires prospective validation to confirm its usefulness and improve its calibration.

Several limitations need to be highlighted in the study performed. First of all, the patients were included before the RECOVERY trial, and therefore, no patients were under steroids during the time of the study.^10^ This could lead to a bias in the estimation of the mortality. Moreover, this study defined the patients aged above 60 years, while some authors recommend to use a cut-off 65 or even 75 years old, the World Health Organization define aging with an age over 60 years old.^11,12^ Finally, the number of patients included limits the generality of the results and requires external validation in further study. 

## Conclusion

We provided a useful tool to the clinician to estimate predicted 30-day mortality among the old patient with 4 clinically relevant variables (severity, autonomy, age, and dementia). This tool needs to be validated in further study.

### Declaration of Interest:

The authors have no conflict of interest to declare.

## Figures and Tables

**Table 1.** Descriptive Statistics

	D30 Survivor (n = 171)	D30 Non-Survivor (n = 60)	*P*	Missing Variable (%)
Age (years)	72 [66-76]	78 [71-82]	<.001	*0 (0.0)*
Sex (male)	127 (74.3%)	47 (78.3%)	.650	* 0 (0.0)*
BMI	27 [24-30]	27 [24-32]	*.575*	*38 (16.4)*
Dementia	2 (1.2%)	8 (13.3%)	<.001	*0 (0.0)*
Frailty (Fried score ≥2)	16 (9.4%)	18 (30.0%)	<.001	*0 (0.0)*
IADL score	8 [8-8]	6 [4-8]	<.001	*4 (1.7)*
Fall in 6 months before	15 (8.8%)	15 (25.0%)	.003	*0 (0.0)*
Recent weight loss	30 (25.9%)	9 (20.5%)	.613	71 (30.7)
CIRS	5 [2-7]	7 [4-11]	<.001	*0 (0.0)*
**Severity at ICU admission**				
Delay between symptom and ICU	10 [6-13]	6 [4-9]	<.001	16 (6.9)
Hospitalization prior to ICU admission	97 (57.1%)	38 (64.4%)	.404	2 (0.9)
SAPS II	37 [32-46]	47 [39-56]	<.001	6 (2.6)
PaO_2_/FiO_2_ ratio during admission	124 [91-160]	109 [80-150]	.111	27 (11.7)
**Biological variable at ICU admission**				
Leucocytes g L_-1_	7.9 [5.9-10.6]	8.4 [5.8-10.6]	.849	21 (9.1)
Neutrophil g L_-1_	6.5 [4.5-8.9]	6.3 [4.4-8.4]	.504	13 (5.6)
Lymphocytes g L_-1_	0.8 [0.5-1.0]	0.7 [0.5-1.2]	.284	58 (25.1)
Monocytes g L_-1_	0.3 [0.2-0.5]	0.3 [0.2-6.5]	.953	28 (12.1)
Platelets g L_-1_	213 [179-298]	199 [146-266]	.065	20 (8.7)
Hemoglobin g dL_-1_	12.5 [11.3-13.9]	12.4 [10.5-13.7]	.298	31 (9.1)
CRP mg L_-1_	160 [108-233]	150 [90-204]	.322	77 (33.3)
Procalcitonin µg L_-1_	0.50 [0.19-1.25]	0.69 [0.33-1.86]	.138	88 (38.1)
Creatinine µmol L_-1_	79 [62-96]	92 [68-159]	.004	5 (2.2)

Results are expressed as the number of patients (n) and percentage (%) or median and IQR. *P* values for the comparison between groups, except for the matching parameters. CRP, C-protein reactive; IQR, interquartile range; CIRS, Cumulative Illness Rating Scale; ICU, intensive care unit. P values less than .05 were considered significant.

**Table 2.** Odds Ratio of the Logistic Regression

	Odds Ratio	CI	*P*
IADL score (point)	0.82	0.71-0.94	*.004*
Dementia	6.22	1.00-38.58	.049
Age (year)	1.12	1.07-1.18	<.001
SAPS II (point)	1.05	1.02-1.08	<.001
